# Supplementary material for: Relationship between age at menarche and metabolic diseases in Korean postmenopausal women: The Korea National Health and Nutrition Examination Survey 2016–2018
Source: PLoS One. 2023 Jan 25;18(1):e0280929. doi: 10.1371/journal.pone.0280929 (PMC9876285; doi:10.1371/journal.pone.0280929)
Supplement: Attachment — Submitted filename: Response to Reviewers.docx [file pone.0280929.s001.docx]

PONE-D-22-21289

Relationship between age at menarche and metabolic diseases in Korean postmenopausal women: The Korea National Health and Nutrition Examination Survey 2016-2018

PLOS ONE

Dear Dr. Lee,

Thank you for submitting your manuscript to PLOS ONE. After careful consideration, we feel that it has merit but does not fully meet PLOS ONE’s publication criteria as it currently stands. Therefore, we invite you to submit a revised version of the manuscript that addresses the points raised during the review process.

==============================

ACADEMIC EDITOR:

I performed the first reading on your work, it seems to be very promising, however, so that we can proceed with the peer analysis, there is a need to adjust the format of the citations, and to review the best formatting for table 1.

-> Thank you for your kind advice. As you recommended, we change the format of the citations and table.
