# Supplementary material for: Relationship between age at menarche and metabolic diseases in Korean postmenopausal women: The Korea National Health and Nutrition Examination Survey 2016–2018
Source: PLoS One. 2023 Jan 25;18(1):e0280929. doi: 10.1371/journal.pone.0280929 (PMC9876285; doi:10.1371/journal.pone.0280929)
Supplement: Attachment — Submitted filename: Response to Reviewers.docx [file pone.0280929.s002.docx]

PONE-D-22-21289

Relationship between age at menarche and metabolic diseases in Korean postmenopausal women: The Korea National Health and Nutrition Examination Survey 2016-2018

PLOS ONE

Dear Dr. Lee,

Thank you for submitting your manuscript to PLOS ONE. After careful consideration, we feel that it has merit but does not fully meet PLOS ONE’s publication criteria as it currently stands. Therefore, we invite you to submit a revised version of the manuscript that addresses the points raised during the review process.

==============================

ACADEMIC EDITOR:

I performed the first reading on your work, it seems to be very promising, however, so that we can proceed with the peer analysis, there is a need to adjust the format of the citations, and to review the best formatting for table 1.

-> Thank you for your kind advice. As you recommended, we changed the format of the citations and table.

PONE-D-22-21289R1

Relationship between age at menarche and metabolic diseases in Korean postmenopausal women: The Korea National Health and Nutrition Examination Survey 2016-2018

PLOS ONE

Dear Dr. Lee,

Thank you for submitting your manuscript to PLOS ONE. After careful consideration, we feel that it has merit but does not fully meet PLOS ONE’s publication criteria as it currently stands. Therefore, we invite you to submit a revised version of the manuscript that addresses the points raised during the review process.

==============================
ACADEMIC EDITOR: Glad you got back in time with some of the requested changes. However, the paper lacks attention, with regard to the format of the citations, where I invite you to review them, following the regulations by the International Committee of Medical Journal Editors (ICMJE).
==============================

-> We apologize for not clarifying this point. Reference number 16 was changed from the website to a more accurate journal. Also, we remove the Endnote code and convert to plain text.
